# Chloroform in Intermittent Fever

**Published:** 1868-02

**Authors:** D. Scott

**Affiliations:** Bellefontaine, Iowa


ARTICLE V.

### CHLOROFORM IN INTERMITTENT FEVER.

BELLEFONTAINE, Iowa, Dec. 19th, 1867.

PROFESSOR N. S. DAVIS,

*Dear Sir:*—In glancing over some back numbers of the MEDICAL EXAMINER, my attention was attracted by an article on the use of chloroform in intermittents, from the pen of Dr. George F. Brickett, of Chicago. The result of the Dr.'s experience is so different from mine, that I have thought proper to drop you a few lines on the subject.

Practising in a country watered by the Des Moines and its numerous tributaries, I have had, during my residence here, ample opportunity to witness malarious disease in all its various phases, from the simple tertian to the most malignant typho-malarial fever, in consequence of which few medicinal agents recommended for this class of disease have escaped my attention. The most remarkable feature of Dr. Brickett's cases was their prompt recovery without the use of the salts of quinia or their succedaneum. I would not be understood to cast the remotest suspicion on the truth of that gentleman's statement; but, on the contrary, I believe his article to be a plain statement of facts, confirmed by the observation of others; but with me the result has been different. During the past summer and autumn, I have administered chloroform, and carefully noted its effect, in upwards of fifty cases of ague, with the following results: in twenty cases, after the administration of one fluid drachm each, the chill was immediately arrested, with the exception of one case, in which the above dose was repeated in one hour; in eleven of the above cases, the febrile stage was probably abridged; of the remaining cases, the fever ran about as usual, all, with few exceptions, terminating in profuse perspiration; in eight of the cases, the paroxysm returned on the succeeding day, and nine on the second day, and three escaped, but were subsequently attacked in from seven to twenty days; of the remaining cases, no reliance was placed in the curative properties of the chloroform (which I only administered for the purpose of abridging the chill), but was followed by large doses of sul. quinia as soon as the sweating stage was established.

In conclusion, allowing my experience to be the guide, I would say that chloroform is a valuable and safe hypnotic; administered in doses of one fluid drachm, in the cold stage of an intermittent, it will never fail to arrest the chill in from ten to fifteen minutes, the patient falling into a refreshing slumber, as described by Dr. Brickett. Exceptionally, the above dose may have to be repeated in from one-half to one hour, but never oftener, and should never be administered where much nausea exists, for it will frequently produce the most distressing vomiting, the fumes of the chloroform regurgitating through the mouth and nostrils, almost threatening the patient with suffocation. I administered the chloroform undiluted, following immediately with cold water. Very truly, etc.,

D. SCOTT.

